# Minimally invasive monitoring of CD4 T cells at multiple mucosal tissues after intranasal vaccination in rhesus macaques

**DOI:** 10.1371/journal.pone.0188807

**Published:** 2017-12-08

**Authors:** Stephanie Dorta-Estremera, Pramod N. Nehete, Guojun Yang, Hong He, Bharti P. Nehete, Kathryn K. Shelton, Michael A. Barry, K. Jagannadha Sastry

**Affiliations:** 1 The University of Texas MD Anderson Cancer Center, Department of Immunology, Houston, TX, United States of America; 2 The University of Texas MD Anderson Cancer Center, Department of Veterinary Sciences, Bastrop, TX, United States of America; 3 The University of Texas Graduate School of Biomedical Sciences at Houston, Houston, TX, United States of America; 4 The University of Texas MD Anderson Cancer Center, Department of Stem Cell Transplantation, Houston, TX, United States of America; 5 Mayo Clinic, Department of Internal Medicine, Division of Infectious Diseases, Rochester, MN, United States of America; 6 Mayo Clinic, Department of Molecular Medicine, Rochester, MN, United States of America; 7 Mayo Clinic, Department of Immunology, Rochester, MN, United States of America; 8 Mayo Clinic, Translational Immunology Virology and Biodefense Program, Rochester, MN, United States of America; University of Nebraska Medical Center, UNITED STATES

## Abstract

Studies in nonhuman primates (NHP) for prospective immune cell monitoring subsequent to infection and/or vaccination usually rely on periodic sampling of the blood samples with only occasional collections of biopsies from mucosal tissues because of safety concerns and practical constraints. Here we present evidence in support of cytobrush sampling of oral, rectal, and genital mucosal tissues as a minimally invasive approach for the phenotypic analyses of different T cells subsets de novo as well as prospectively after intranasal immunization in rhesus macaques. Significant percentages of viable lymphocytes were obtained consistently from both naïve and chronically SIV-infected rhesus macaques. The percentages of CD3+ T cells in the blood were significantly higher compared to those in the mucosal tissues analyzed in the naïve animals, while in the SIV+ animals the CD3+ T cells were significantly elevated in the rectal tissues, relative to all other sites analyzed. In the naïve, but not SIV+ macaques, the rectal and vaginal mucosal tissues, compared to oral mucosa and blood, showed higher diversity and percentages of CD4+ T cells expressing the HIV entry co-receptor CCR5 and mucosal specific adhesion (CD103) as well as activation (HLA-DR) and proliferation (Ki67) markers. Sequential daily cytobrush sampling from the oral, rectal, and genital mucosal tissues was performed in SIV+ animals from an ongoing study where they were administered intranasal immunization with adenoviral vectored vaccines incorporating the green fluorescent protein (GFP) reporter gene. We detected a transient increase in GFP+ CD4 T cells in only oral mucosa suggesting limited mucosal trafficking. In general, CD4+ and CD8+ T cells expressing Ki67 transiently increased in all mucosal tissues, but those expressing the CCR5, HLA-DR, and CD103 markers exhibited minor changes. We propose the minimally invasive cytobrush sampling as a practical approach for effective and prospective immune monitoring of the oral-genital mucosal tissues in NHP.

## Introduction

Worldwide, the majority of infections by the human immunodeficiency virus (HIV) are acquired through mucosal surfaces [1]. Thus, it is important to understand the immune cell repertoire at the mucosal tissues, specifically CD4+ T cells that serve as the primary targets of HIV infection and as central players of the cellular immune responses [2, 3]. Furthermore, central to understanding the immune responses occurring at mucosal sites post-vaccination or infection is the need for detailed analyses of activated CD4+ T cells and those expressing markers implicated in mucosal homing and susceptibility to HIV/SIV infection. Serial sampling via biopsies is impractical, causes discomfort to the subject, and takes several days for the biopsy site to heal. Cell yields from swabs and lavage collections are generally insufficient for detailed profiling of the phenotype and functions of various immune cell subsets [4]. A recent international multicenter study demonstrated cervical brushing, relative to biopsies as the optimal sampling procedure in human clinical trials for accurately and consistently determining cellular immune responses in the female reproductive tract [5]. Therefore, brushings of mucosal surfaces may provide a non-invasive approach to analyze immune cell subsets at these areas [6].

Taking advantage of an ongoing study, we performed serial cytobrush sampling of the oral, rectal and genital mucosal tissues in a small cohort of chronically SIV-infected rhesus macaques along with matching naïve control animals. Specifically, we analyzed for the distribution of CD4+ and CD8+ T cells subsets in the different mucosal tissues along with those in the blood, and also the kinetics of changes in the T cells subsets after intranasal dosing of SIV+ macaques with recombinant adenoviruses (Ad) expressing HIV/SIV genes as well as GFP and luciferase reporter genes [7, 8]. Data from this investigation strongly support cytobrush sampling as not only a practical approach for effective minimally invasive sampling technique but also for prospective monitoring of the frequencies and phenotypes of immune cells by combining with multi-factorial flow cytometry for effective screening of candidate HIV vaccines in nonhuman primate (NHP) models.

## Materials and methods

### Animals

**Fig 1:**
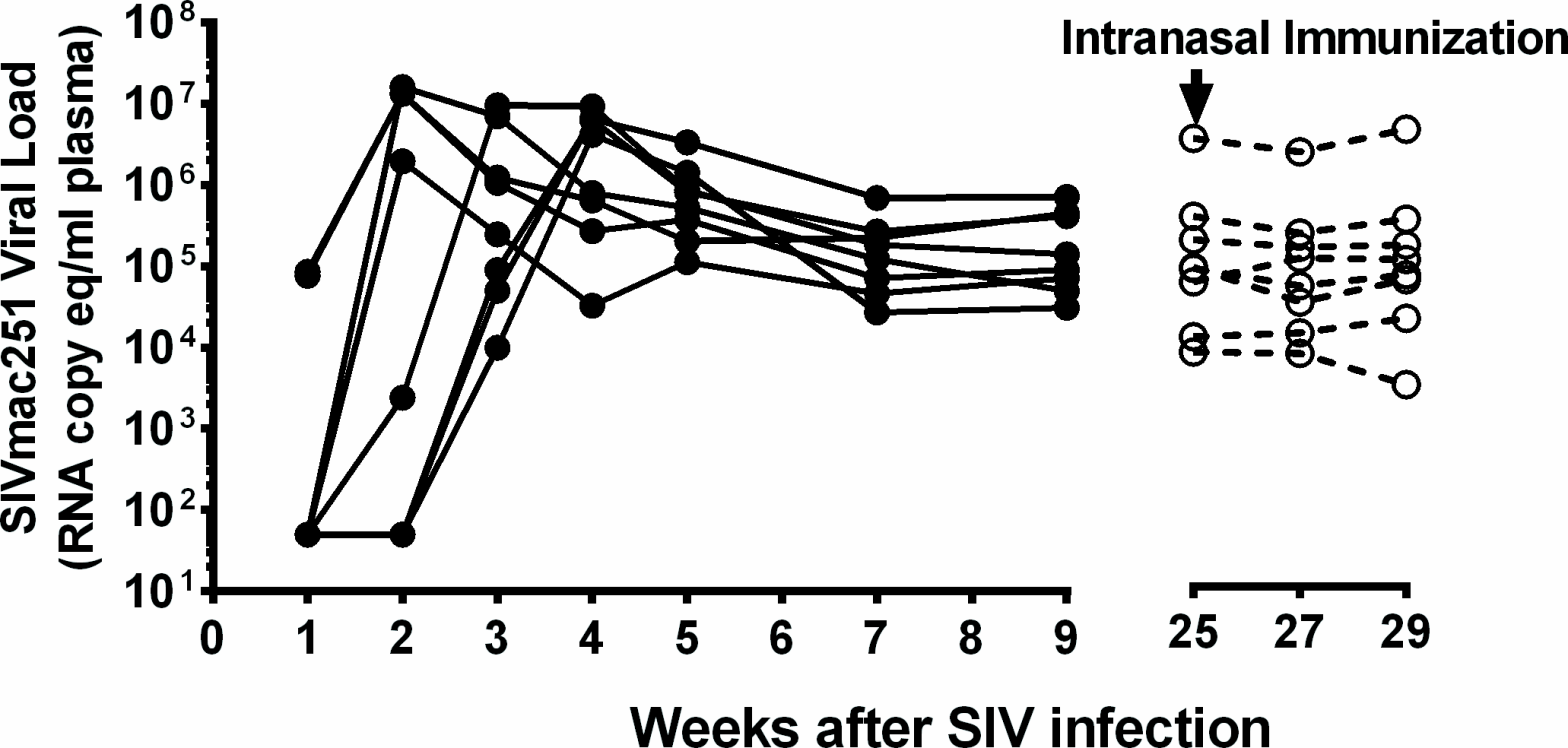
Timeline of SIV challenge, SIV viral loads, and Ad vaccine immunization. Plasma viral loads for individual macaques at different time points after SIVmac251 challenge are shown as RNA copy equivalents/ml. At week 25 the animals were administered intranasal Ad vaccine as described in the methods section and the viral loads were monitored for the next four weeks as shown. These assays were performed at the NIH Core Facility by Dr. Jeff Lifson's group. The threshold sensitivity of the assay is 30 viral RNA copy-equivalents/ml of plasma, and the inter-assay variation is <25% (coefficient of variation).

Studies included both naïve and chronically SIV-infected adult Rhesus macaques (*Macaca mulatta*) of Indian origin at the Michael Keeling Center for Comparative Medicine and Research of The University of Texas MD Anderson Cancer Center, Bastrop TX, housed in chambers that were 44’W x 88’H x 160’D. Experiments were approved by the Institutional Animal Care and Use Committee at the University of Texas MD Anderson Cancer Center and were performed according to the principles of the NIH Guide for the Care and Use of Laboratory Animals, the provisions of the Animal Welfare Act, PHS Animal Welfare Policy, and the policies of the University of Texas MD Anderson Cancer Center. All animals were given water ad libitum and were fed monkey diet (Harlan). Manipulanda, visual stimulation, and auditory stimulation were provided for additional enrichment. Animals were monitored daily. Anesthetics/analgesics were used to minimize discomfort and pain during procedures. As an endpoint, animals were euthanized with ketamine (11 mg/kg) followed by Beuthanasia (1 ml/10 lb). Cytobrush and peripheral blood samples were obtained from naïve and SIV+ animals (n = 7 or 8) that were part of an ongoing vaccine study where they were included in the control un-vaccinated group and were infected with SIV_mac251_ via repeat low-dose rectal challenge. During the two week follow up subsequent to intranasal immunization, for which sequential samples were collected, the females were all in the similar cycling status. At the time of the present investigation, all the animals were chronically infected for 25 weeks after start of SIV challenge as shown in Fig 1.

### Intranasal immunization

A group of eight SIV+ animals (4 males and 4 females) was anesthetized by intravenous injection of ketamine (5–10 mg/kg) and immunized by intranasal route with the animals in dorsal recumbency and the head down [9]. The 100 μl of inoculum containing 10^11^ virus particles (vp) of recombinant adenovirus expressing HIV/SIV genes as well as GFP and luciferase reporter genes was delivered in the nares (50 μl in each nare) using an appropriate sized slip pipette tip. Following inoculation, animals were observed twice daily.

### Collection of samples

Cytobrush and blood samples were collected from naïve animals at a single time point and from SIV+ animals at prior to and after intranasal vaccination daily for 4 days with a final sampling at day 14. Peripheral venous blood samples were collected in EDTA-coated tubes and the mononuclear cells, PBMCs were prepared from the blood using the standard Ficoll-Hypaque density gradient centrifugation. Subsequently, the cells were washed twice with RPMI 1640 medium supplemented with 5 mM glutamine, amphotericin B, penicillin, streptomycin, and 10% fetal bovine serum (FBS) [10]. Samples with cell viability ≥90%, assessed by the trypan blue exclusion method, were used in flow cytometry analyses. Mucosal cytobrush samples from oral cavity, rectum and vagina/urethra were collected from anesthetized animals with a disposable sterile cytobrush Plus GT (Medscand Medical, Cooper surgical company, Trumbull, CT, USA) as described with modification [11]. Briefly, the mucosal surface was cleaned of excess mucus and exudate. The oral sample is collected by swiping and rolling the cytobrush 2–3 times against the mucosa of the cheek pouch on both sides of the mouth and against the base of the tongue. The rectal cytobrush sample is collected by first manually extracting the feces from the rectum with a gauze-covered finger if needed. The cytobrush is then swiped and rolled against the rectal mucosa 2–3 times. The vaginal cytobrush samples are collected by gently inserting the cytobrush into the vagina approximately 2 cm being careful to avoid traumatizing the cervix. The brush is then swiped and rolled against the vaginal mucosa 2–3 times. The urethral samples from male monkeys are collected using a micro brush. The head of the penis is gently grasped by the fingers and extended so the penis and urethra are stabilized in a straight extended position. The brush is inserted into the urethra approximately 2 cm and gently swiped and rolled against the urethral mucosa. Immediately after the collection procedure, each cytobrush is placed in 2 mL of cold transport medium (RPMI 1640 medium supplemented with 5mM glutamine, amphotericin B, penicillin, streptomycin, and 10% FBS). Cells were isolated within 4 h of collection by gently rotating the cytobrush against the sides of the tube to dislodge cells. The transport medium was flushed through the cytobrush bristles 5 times by using a sterile pipette to dislodge all sample-derived cells. The cell suspension was transferred to a sterile 15-ml centrifuge tube, and the cells were pelleted at 250 ×*g* for 10 min and resuspended in 2 ml of 10% FBS RPMI (transport medium) for use in flow cytometry analysis.

### Flow cytometry

**Fig 2:**
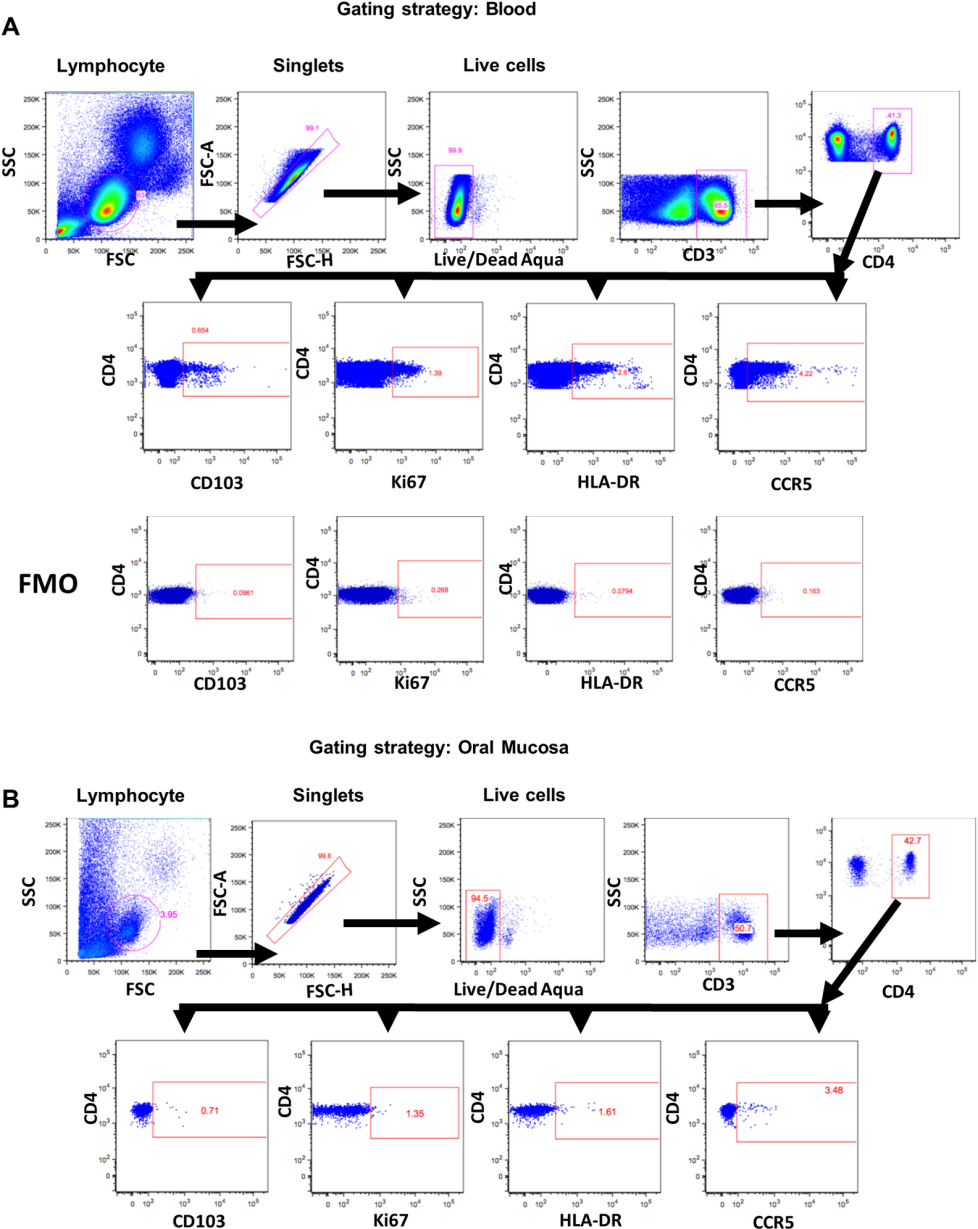
Gating strategy for the identification of T cells subsets from blood and mucosal cytobrush samples. Representative gating strategy to analyze for CD3+ T cells and the CD4+ T cells subsets expressing surface (CCR5, CD103, HLA-DR) and intracellular markers (Ki67) from (A) blood and (B) oral cytobrush. (A, bottom panel) A representative plot with the gating of FMO controls for CD103, Ki67, HLA-DR and CCR5 performed on PBMCs is shown.

**Fig 3:**
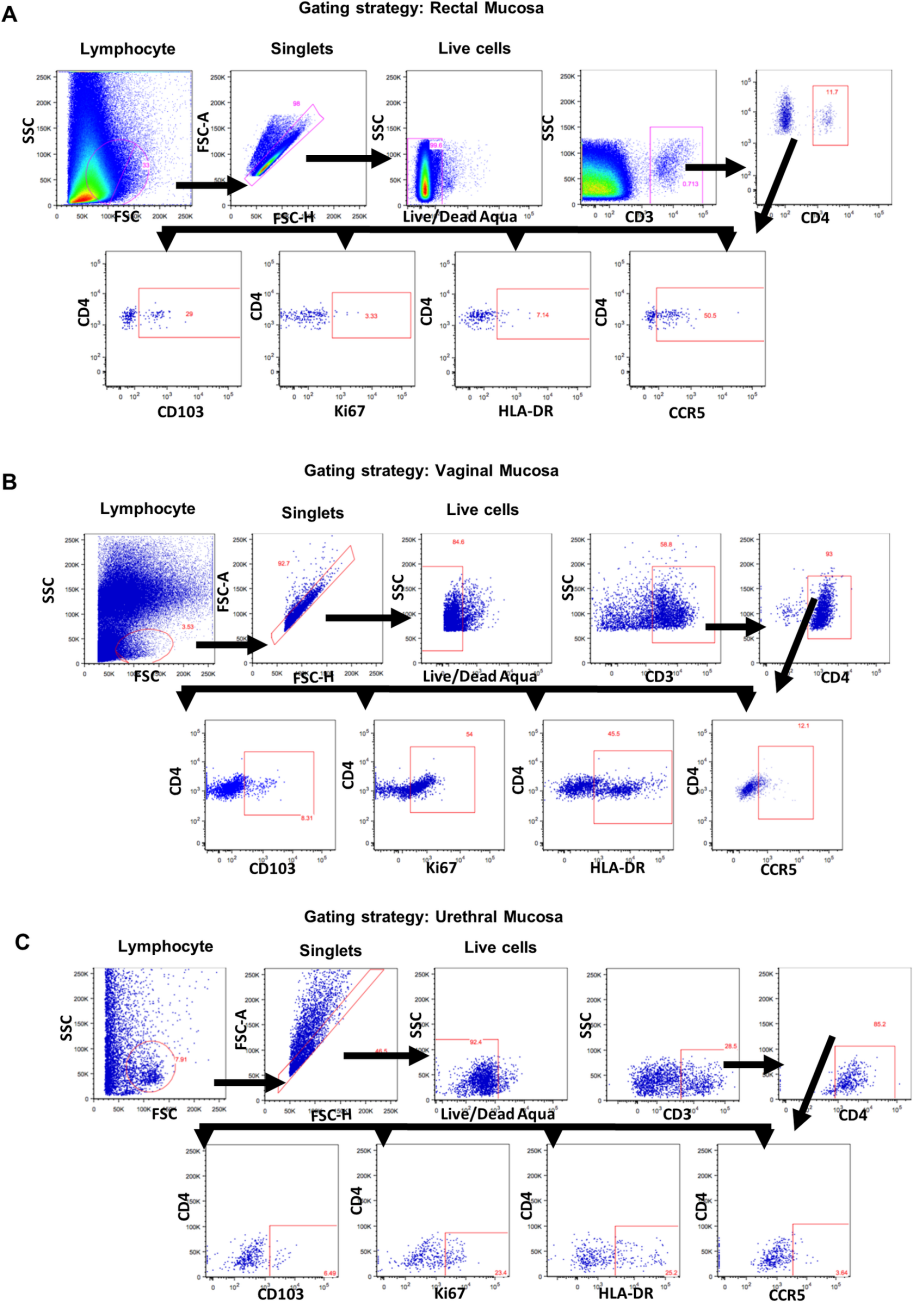
Gating strategy for the identification of T cells subsets from blood and mucosal cytobrush samples. Representative gating strategy to analyze for CD3+ T cells and the CD4+ T cells subsets expressing surface (CCR5, CD103, HLA-DR) and intracellular markers (Ki67) from (A) rectal, (B) vaginal and (C) urethral cytobrushes.

Cells collected with the cytobrush from oral, rectal, vaginal/penile tissues were washed twice with sterile PBS and along with PBMC were used for T cell phenotyping. Because of the smaller group size of 8 animals with 4 each of males and females, data for the vaginal and urethral cytobrush samples were plotted and analyzed together and shown as genital mucosal samples. Aliquots of cells were incubated on ice for 45 min with a panel of human antibodies that cross-react with rhesus macaque samples The panel included antibodies against human CD3 (clone SP34-2, PE-Cy7-labeled), CCR5 (clone 3A9, PE), Ki67 (clone B56, PerCP-Cy5.5-labeled); and HLA-DR (clone G46-6, PE-Cy5-labeled) all from BD Bioscience (San Jose, CA); CD4 (clone OKT4, Pacific Blue-labeled) from ThermoFisher Scientific (Waltham, MA); and CD103 (clone 2G5, APC-labeled) from Beckman Coulter (Indianapolis, IN). Dilutions for antibodies were determined by following manufacturer’s recommendations. Dead cells were excluded by using live-dead fixable dead cells stain kit obtained from Invitrogen (Carlsbad, CA). Subsequently, the cells were washed twice with PBS containing 2% FBS and then fixed and permeabilized with FoxP3 Fix/Perm Kit (ThermoFisher Scientific, Waltham, MA). The nuclear marker Ki67 was stained with an antibody specific for Ki67 in permeabilization buffer. Compensation controls (OneComp eBeads, (ThermoFisher Scientific, Waltham, MA) were prepared and used during the setup of the flow cytometer. Gating was performed by using fluorescence minus one (FMO) controls (in PBMCs) and by using internal negative controls for cytobrushes to prevent splitting of the samples (Fig 2A). All the samples were collected on an LSR II instrument (BD Biosciences, San Jose, CA) and were analyzed using FlowJo software (FlowJo, LLC, Ashland, Oregon). Approximately 2 x 10^5^ to 1 x 10^6^ live total cell events were collected per sample. The gating strategy for detecting the T cells expressing the different surface and intracellular markers for PBMCs (Fig 2A), oral mucosa (Fig 2B), rectal mucosa (Fig 3A), vaginal mucosa (Fig 3B) and urethral mucosa (Fig 3C) are shown.

### Data analysis

Graphs and statistical analyses were performed using Prism Graphical software. For intra-individual differences in the various immune cell subsets, we performed repeated measures one-way ANOVA with Greenhouse-Geisser correction and Tukey’s multiple comparison test. For the analyses of sequential samples post-immunization, only groups that showed significant differences compared to day 0 were marked. To analyze for differences in various immune cell subsets between naïve and SIV-infected macaques, we used ordinary 2-way ANOVA with Tukey’s multiple comparisons test.

## Results

### Phenotypic analysis of T cells subsets from the blood and oral-genital mucosal tissues of naïve and chronically SIV-infected rhesus macaques

Overall, the numbers of live lymphocytes isolated from the different mucosal tissues using the cytobrushes were amenable for flow cytometry analysis of the different lymphocyte subsets (Table 1). To determine the percentages of different T cells subsets in blood and the different mucosal sites we used the gating strategy shown in Fig 2 and Fig 3.

**Table 1:** Cell yields from cytobrushes at different mucosal compartments from naïve and SIV-infected macaques.

Cytobrushes from Naïve			Cytobrushes from Infected	
Tissue	% Live Lymphocytes	Live lymphocyte count	% Live Lymphocytes	Live lymphocyte count
Oral	54.3 (17.1–57.02)	789 (188–2051)	87.3% (68.3–97.4)	12925 (1801–48948)
Rectal	79 (43.9–99.7)	3860 (1765–239875)	67.3 (17.7–92.8)	36781 (2069–606000)
Urethral	42.8 (31–54)	297 (192–402)	71.6 (30.1–94.4)	8199 (787–359000)
Vaginal	32.8 (20.4–46.1)	1136 (451–945)	63.3 (39.6–85.5)	5578 (739–124000)

**Fig 4:**
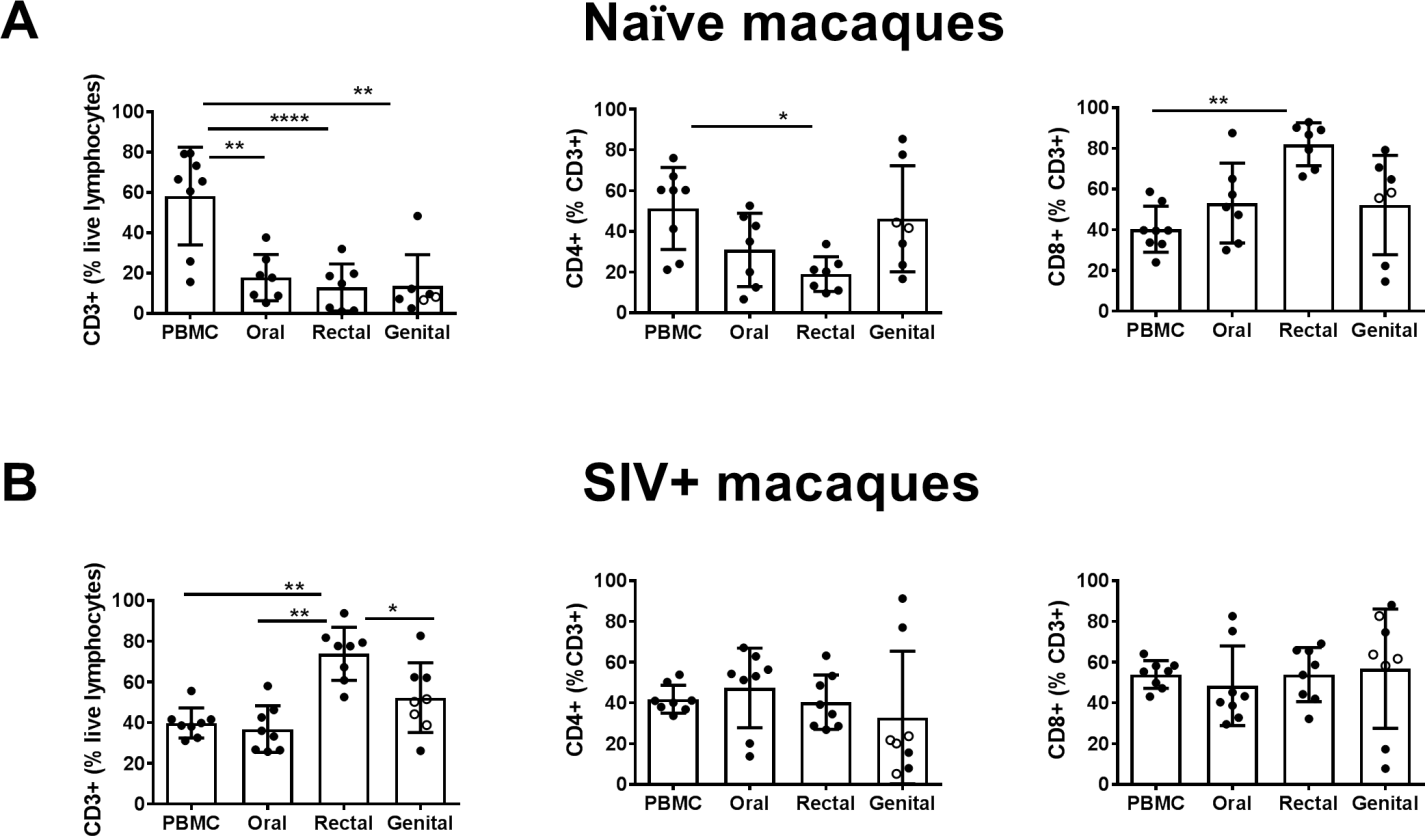
T cell composition in the blood and different mucosal compartments in naïve and SIV+ macaques. Cells from PBMCs or oral, rectal, or genital cytobrushes were collected and the percentage of CD3+ among lymphocytes (left panel), the percentage of CD4+ T cells among CD3+ cells (middle), and the percentage of CD3+,CD4- (CD8+) cells (right panel) were quantified in (A) naïve macaques and (B) SIV+ macaques. Ordinary two-way ANOVA was performed to identify statistical differences. Data points for the genital tissue samples from male and female animals representing urethral and vaginal cytobrushings were represented with open and closed symbols, respectively.

In the naïve animals, we observed significantly lower frequency of CD3+ T cells at the different mucosal compartments compared to those in the PBMCs (Fig 4A). The percentages of CD4+ T cells were comparable between blood and mucosal tissues except those in the rectal mucosa which was significantly lower; while the CD8+ T cell percentages were significantly higher in the rectal mucosa relative to that in blood as well as other mucosal sites. In contrast, analyses of T cells subsets in the chronically SIV-infected macaques revealed significantly higher percentages of CD3+ T cells within the rectal mucosa relative to all other mucosal tissues and the blood, while the CD4+ and CD8+ T cells subsets were evenly distributed among all the sites analyzed (Fig 4B).

**Fig 5:**
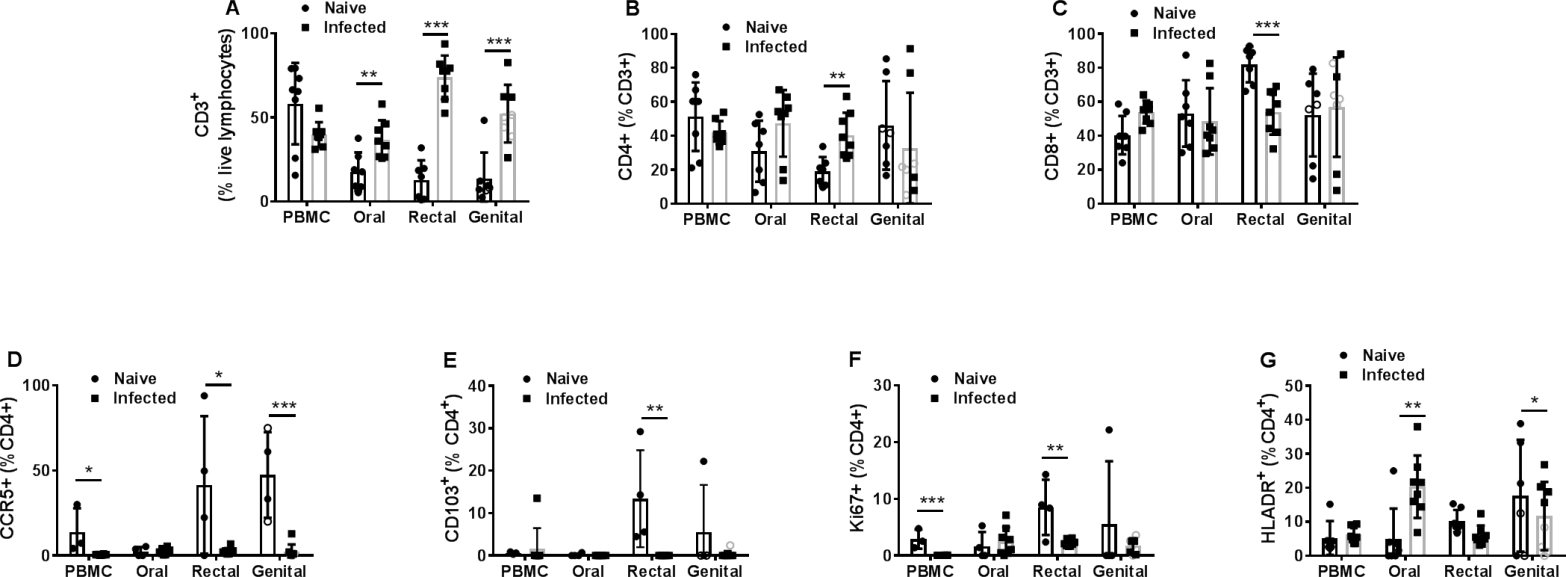
Analyses of T cells subsets in naïve and SIV-infected macaques. Cells from PBMCs or oral, rectal, or genital cytobrushes were collected and the percentages of (A) CD3+, (B) CD4+, and (C) CD3+,CD4- (CD8+) in naïve and SIV+ macaques were quantified. The percentages of (D) CCR5+, (E) CD103+, (F) Ki67+, and (G) HLA-DR+ populations among CD4+ T cells in naïve and infected macaques are shown. (A–E) Data represented as means ± SD (n = 3–8). *P* values: *p<0.05, ** *p*<0.005, ***p<0.0005. Two-way ANOVA was performed to detect statistical difference between groups. Genital mucosal cell analyses included cytobrush sampling from urethra in males (open symbols) and vagina in females (closed symbols).

Further comparative analyses of the T cell subsets between naïve and the SIV+ macaques identified differences in T cell populations at the mucosal areas in infected macaques. The percentages of CD3+ T cells in the SIV+ animals, relative to those in naïve animals were significantly higher at all three mucosal sites (Fig 5A). The percentages of the CD4+ and CD8+ T cells subsets were comparable between the naïve and SIV+ macaques except in the rectal tissues of the SIV+ animals where the CD4+ T cells were significantly higher but CD8+ T cells were significantly lower relative to naïve animals (Fig 5B and 5C). We also observed that CD4+ T cells expressing the HIV co-receptor CCR5, as well as the proliferation (Ki67), and mucosal homing integrin (CD103) markers were significantly reduced in blood and most mucosal tissues in infected macaques relative those in the naïve animals (Fig 5D and 5F). On the other hand, the percentage of CD4+ T cells expressing the activation marker HLA-DR was significantly higher in oral mucosa in infected macaques compared to that in naïve animals (Fig 5G).

### Kinetics of changes in CD4+ and CD8+ T cells at oral and genital mucosal tissues after intranasal administration of recombinant adenovirus vaccine in chronically SIV-infected rhesus macaques

Having established the feasibility of using the cytobrushing methodology for T cell profiling at the mucosal tissues, we investigated its applicability for prospective immune monitoring after vaccination. For this, we took advantage of an ongoing study where a group of eight rhesus macaques consisting of 4 males and 4 females exhibiting chronic SIV infection subsequent to repeat low-dose rectal SIVmac251 challenge. At 25 weeks, when the animals were scheduled to be euthanized, the study was extended by 2 weeks to allow the animals to be used in a final study to test the effects of mucosal vaccination on mucosal CD4+ T cell biology.

**Fig 6:**
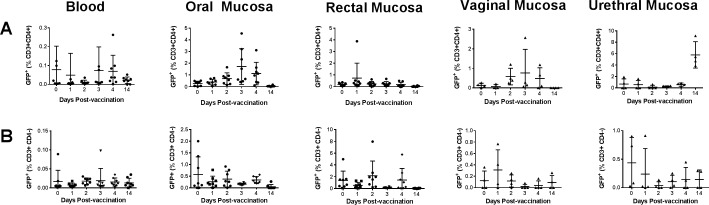
Monitoring of adenovirus-infected cells collected at different time points in the blood and mucosal tissues. Using the GFP reporter gene expression, the percentages of GFP+ cells among (A) CD4+ and (B) CD8+ (CD3+CD4-) T cells were quantified at different time points after vaccination in the blood and mucosal tissues as shown. (A—B) Data represented as means ± SD (n = 8). One-way ANOVA was performed to detect statistical difference between groups.

The animals were immunized with recombinant Ad expressing GFP-Luciferase, clade C HIV envelope gp140, and SIV gag-pol by the intranasal route as described in the methods section. Mucosal cytobrush samples were collected daily after vaccination for 4 days and a final sample on day 14, to determine if the activity of the Ad could be detected in CD3+ CD4+ as well as CD3+ CD4- (considered to be CD8+) T cells at the oral, rectal, urethral and vaginal sites distant from the intranasal mucosal immunization site. In the oral mucosa, closest to the intranasal immunization site, we observed a transient, but nonsignificant increase in the percentages of GFP+ CD4+ T cells at 4d relative to 0d, which then returned to base levels by 14d post-immunization (Fig 6A). No significant changes in the levels of GFP+ populations among either CD4 or CD8 T cells were detected in the rectal and genital tissues analyzed (Fig 6A and 6B).

### Changes in CD4+ T cell subsets in the blood and at mucosal tissues after intranasal immunization

**Fig 7:**
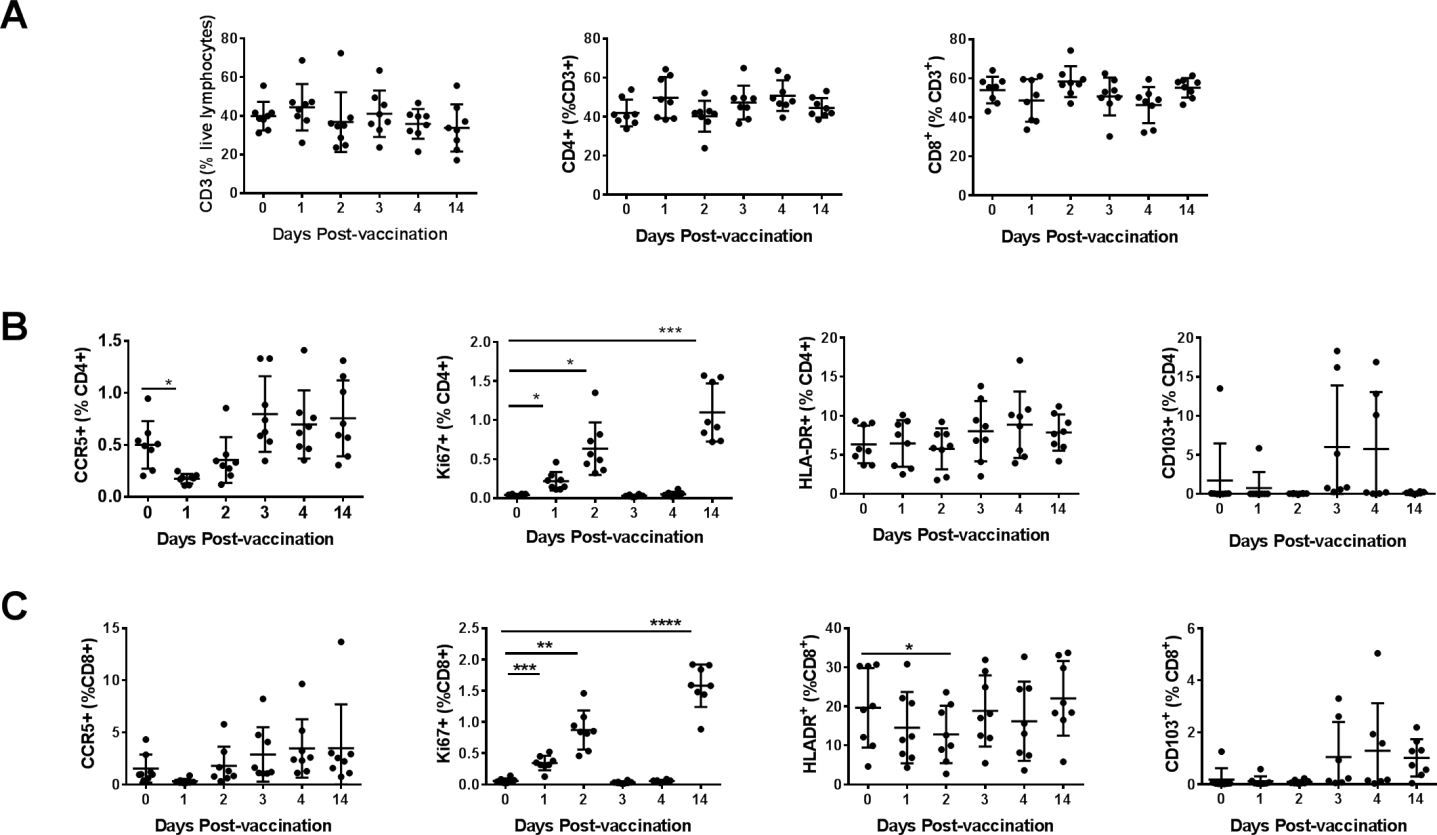
Monitoring changes in T cells subsets after vaccination in the blood. Percentages of different T cell subsets in the blood were quantified at different time points after vaccination. (A) Percentages of CD3+, CD4+, CD8+ (CD3+, CD4-) T cells and, the percentages of CCR5+, Ki67+, HLA-DR+ and CD103+ among (B) CD4+ T cells and (C) CD8+ T cells are shown. Data represented as means ± SD (n = 8). *P* values: *p<0.05, ** *p*<0.005, ****p<0.00005. One-way ANOVA was performed to detect statistical difference between groups.

**Fig 8:**
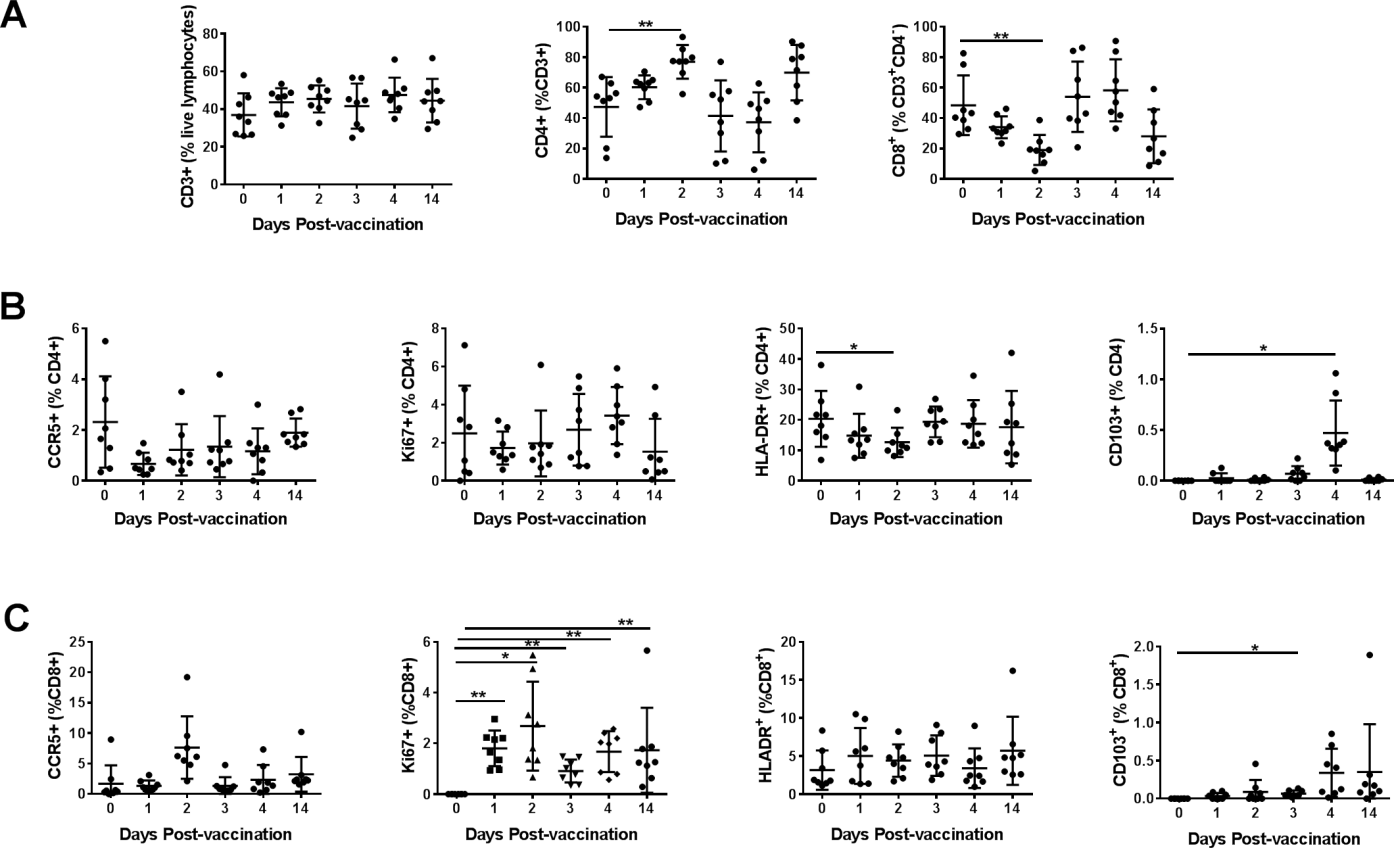
Monitoring changes in T cells subsets after vaccination in the oral mucosa. Percentages of different T cell subsets in the oral mucosa were quantified at different time points after vaccination. (A) Percentages of CD3+, CD4+, CD8+ (CD3+, CD4-) T cells and, the percentages of CCR5+, Ki67+, HLA-DR+ and CD103+ among (B) CD4+ T cells and (C) CD8+ T cells are shown. Data represented as means ± SD (n = 8). *P* values: *p<0.05, ** *p*<0.005. One-way ANOVA was performed to detect statistical difference between groups.

**Fig 9:**
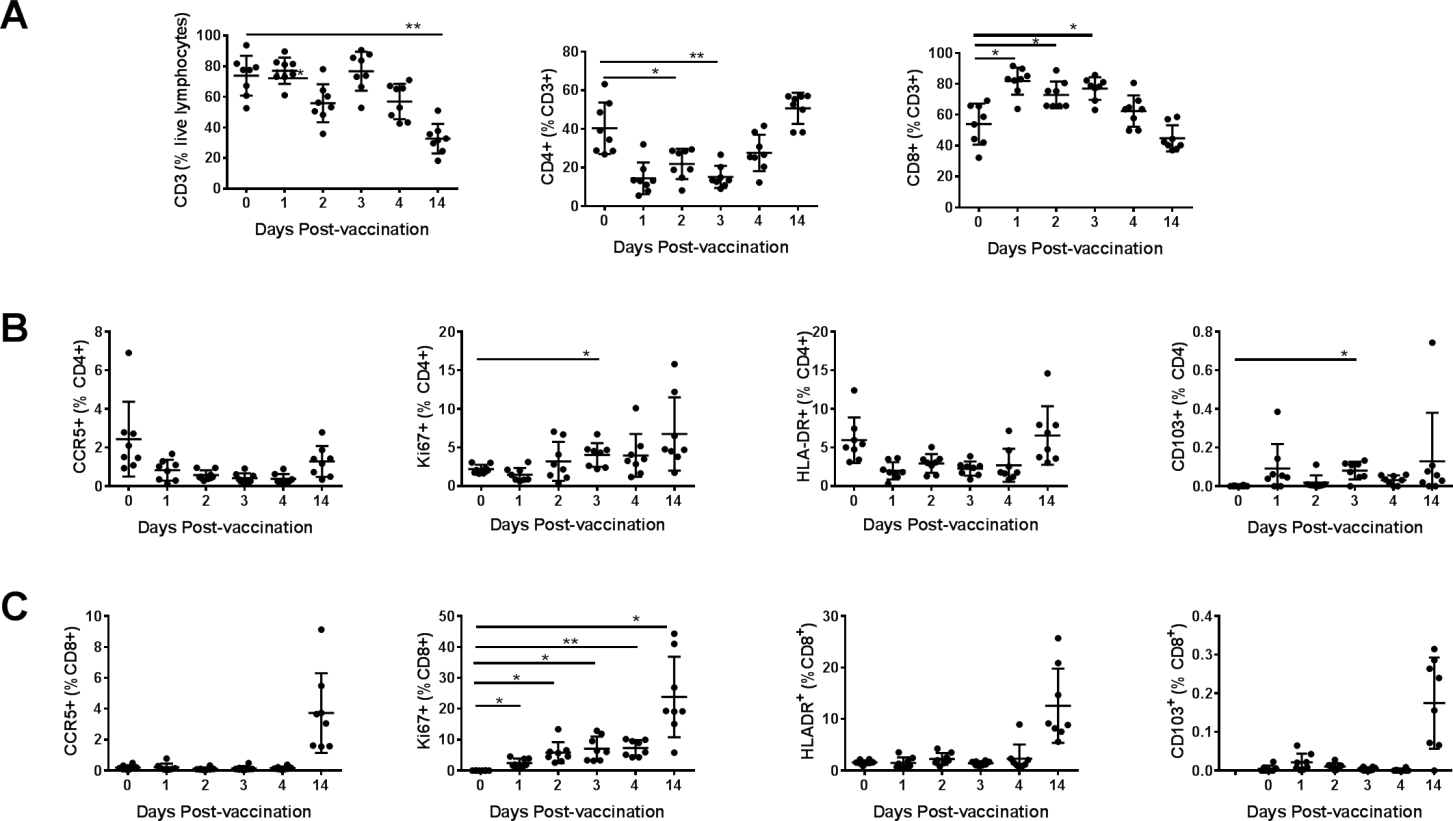
Monitoring changes in T cells subsets after vaccination in the rectal mucosa. Percentages of different T cell subsets in the oral mucosa were quantified at different time points after vaccination. (A) Percentages of CD3+, CD4+, CD8+ (CD3+, CD4-) T cells and, the percentages of CCR5+, Ki67+, HLA-DR+ and CD103+ among (B) CD4+ T cells and (C) CD8+ T cells are shown. Data represented as means ± SD (n = 8). *P* values: *p<0.05, ** *p*<0.005. One-way ANOVA was performed to detect statistical difference between groups.

**Fig 10:**
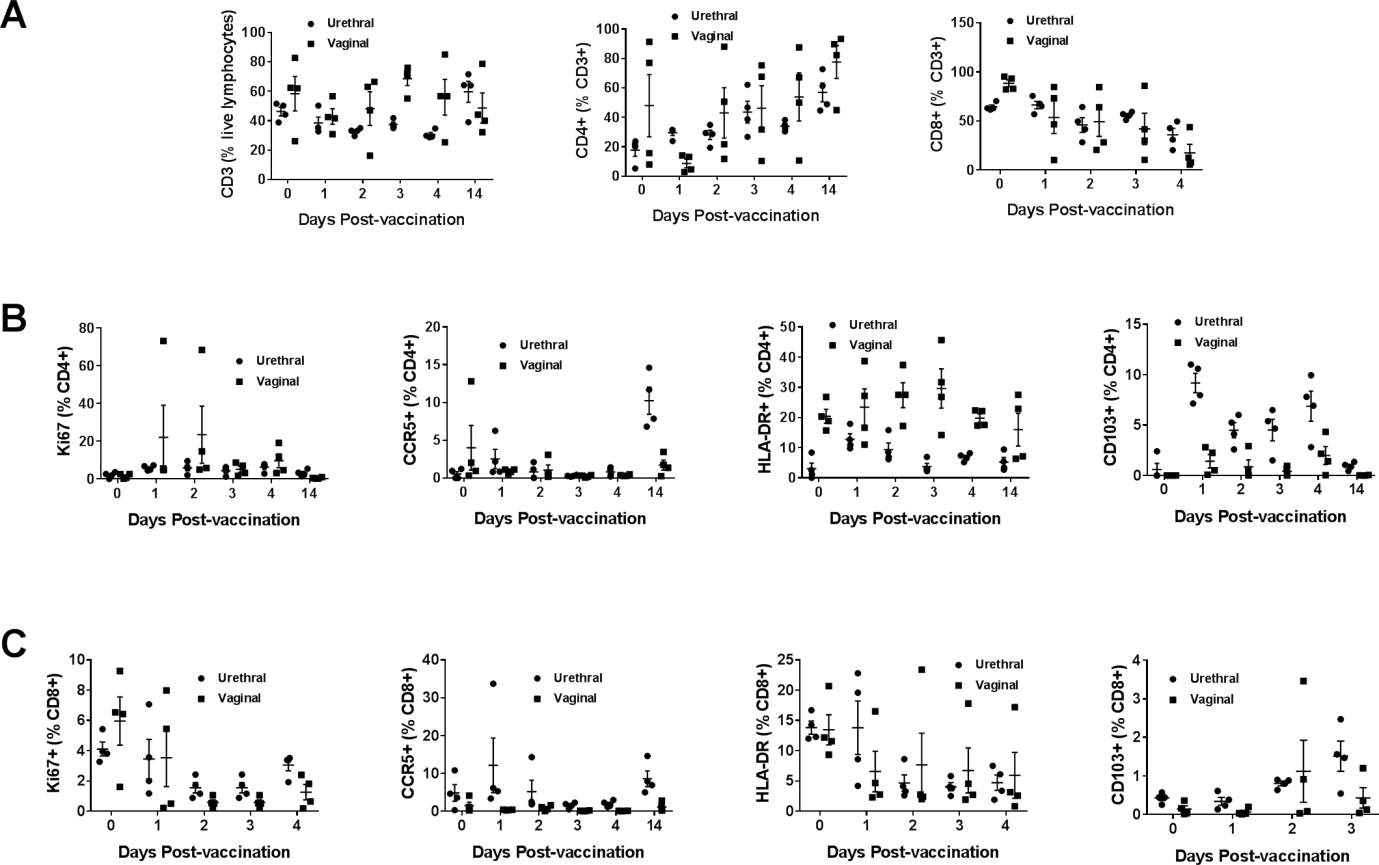
Monitoring changes in T cells subsets after vaccination in the genital mucosa. Percentages of different T cell subsets in the urethral (●) and vaginal (■) mucosa were quantified at different time points after vaccination. (A) Percentages of CD3+, CD4+, CD8+ (CD3+, CD4-) T cells and, the percentages of CCR5+, Ki67+, HLA-DR+ and CD103+ among (B) CD4+ T cells and (C) CD8+ T cells are shown. Data represented as means ± SD (n = 8).

Lymphocytes from blood and the cytobrush samples from different mucosal areas collected on the indicated days were also analyzed for the CCR5, HLA-DR, CD103, and Ki67 markers on the CD4+ and CD8+ T cells using a similar gating strategy as in Fig 2 and Fig 3. Overall, we observed in the blood no significant changes for the percentages of total T cells or CD4+ and CD8+ T cell subsets (Fig 7A). Significant increases in CD4+, as well as CD8+ T cells expressing the proliferation marker Ki67, were observed in the blood after intranasal immunization. Also, CCR5 expressing CD4+ T cells and HLA-DR expressing CD8+ T cells exhibited significant, but transient decreases (Fig 7B and 7C). In the oral mucosal tissues, there was a transient increase in CD4+ T cells and decrease in CD8+ T cells, respectively at day 2 post-immunization (Fig 8). We also observed that populations of CD8+ T cells expressing Ki67 increased progressively to peak by day 2 post-immunization and remained higher at day 14, the last time point analyzed (Fig 8C). The CD4+ T cells expressing HLA-DR transiently decreased while CD103 expressing CD4+ as well as CD8+ T cells transiently increased in the oral mucosa (Fig 8). In the rectal mucosa, the CD3+ T cells, as well as CD4+ T cells subsets, were significantly reduced after the intranasal immunization, but the CD8+ T cells significantly increased between days 1–3 and returned to base levels by day 14 (Fig 9A). The percentages of CD4+ T cells expressing Ki67 and CD103 increased transiently (Fig 9B), while CD8+ T cells expressing Ki67 progressively showed significant increases after immunization in the rectal mucosa (Fig 9C). Although only 4 each of urethral and vaginal cytobrush samples were obtained, thus limiting the statistical power of the study, an increasing trend for CD4+ T cells was observed subsequent to intranasal immunization in both urethral and vaginal mucosa (Fig 10). A differential expression of activation markers on CD4+ and CD8+ T cells was observed between urethral and vaginal mucosa, which changed over time (Fig 10B and 10C).

## Discussion

To our knowledge, the present investigation demonstrates for the first time the feasibility of using cytobrushing for daily sampling and detailed phenotypic analyses of T cells from the oral, rectal and genital (with limited analyses of vaginal/penile) mucosal tissue sites in rhesus macaques. An international multicenter study by McKinnon et al (2014) compared biopsy versus cytobrushing to demonstrate the advantages of accurately and consistently determining cellular immune responses [5]. However, the intent of our study was not to compare cytobrushing with the biopsy technique but to demonstrate the feasibility of prospective monitoring of immune cells at the mucosal tissues by cytobrushing technology. The sequential cytobrush sampling provides adequate numbers of viable cells allowing for detailed and reliable flow cytometric analyses of the different T cell subsets [12].

For most infectious agents, a vector that generates stronger immune responses is likely to mediate better protection. However, for pathogens that infect immune cells, robust vaccine responses may have untoward effects flipping protection towards increased infection. HIV vaccines, specifically those directed to the mucosal tissues are suspected to suffer this unique vaccine side effect. This has been particularly highlighted in concern with regards to adenovirus-based vaccines in light of the STEP trial and NIH conducted trials [13, 14], even though similar increases in lentiviral infection after vaccination has been recorded using several other viral and DNA-based approaches [15–17] One of the most successful vaccination strategies studied in the SIV-rhesus models employs attenuated forms of SIV [18], but even in this approach, it has been shown that mucosal challenge 15 months post-vaccination protected against infection while challenge 2 weeks after vaccination resulted in superinfection [18]. However, none of these studies analyzed for changes in the T cell populations at any of the mucosal tissues and in general relied on assumptions based on correlations with observations within the systemic compartment (e.g. peripheral blood). In this investigation, we have adapted the minimally invasive cytobrush sampling methodology to show the feasibility of prospectively monitoring T cell populations in multiple mucosal tissues along with blood. The cell yields from the cytobrushes that we obtained were comparable to those from the vaginal mucosa in a previous report from human studies where CD45+ leukocytes were obtained using two cytobrushes as opposed to single cytobrushing in our studies [5]. Although we did not analyze for other immune cells in these samples, it is known that NK cells, innate lymphoid cells, neutrophils, dendritic cells, monocytes and macrophages are modulated during HIV/SIV infections [19]. Prospective monitoring by the cytobrushing methodology may allow for detailed understanding of the roles of these populations during HIV/SIV infections at mucosal sites.

Although we observed that mucosal intranasal vaccination employing an adenovirus vector caused transient changes on Ad-infected or activated CD4+ T cells (Figs 7–10), this immunization did not perturb the on-going SIV infections in macaques (Fig 1). Since the current investigation exclusively relied on taking advantage of the availability of chronically SIV-infected macaques from an ongoing study, it is important to determine the generality of changes in the lymphocytes subsets observed subsequent to vaccination in naïve animals. Furthermore, this study was not intended to nor adequately powered to judge vaccination-specific effects on antigen-specific or Ad-specific immunity. Rather, this investigation primarily assessed the feasibility of frequent minimally sampling of mucosal tissues in rhesus macaques for demonstrating immune monitoring potential. Future studies with a bigger cohort of naïve animals would be necessary to address these important aspects of mucosal immunity after vaccination.

Given that mucosal infections are the major modes of HIV transmission worldwide, knowledge on the inherent as well as infection and/or vaccine-mediated immune cell repertoire at the mucosal tissues is essential for rational vaccine design [20]. The implementation of cytobrush sampling for immune monitoring from the oral-genital mucosal sites is shown not only as feasible in this investigation but also applied in the setting of vaccine delivery to determine the immediate kinetics of changes in the T cells subsets at these sites. By including the phenotypic markers such as CCR5, Ki67, and HLA-DR we obtained data on T cells populations that influence infection acquisition vs protective immunity pertinent for vaccination approaches. With the continued improvements in both quality and sensitivity of the flow cytometry methodology, it is possible to include antigen- and mitogen-specific stimulations to adapt this minimally cytobrush sampling methodology as a practical means for studying the kinetic changes in not only the phenotype but also the functional characteristics of different immune cells at the mucosal tissues. The use of this technique in NHP models to monitor immune responses at mucosal sites may aid in the identification of effective vaccines for HIV.
