# Correction: Minimally invasive monitoring of CD4 T cells at multiple mucosal tissues after intranasal vaccination in rhesus macaques

**DOI:** 10.1371/journal.pone.0191378

**Published:** 2018-01-11

**Authors:** Stephanie Dorta-Estremera, Pramod N. Nehete, Guojun Yang, Hong He, Bharti P. Nehete, Kathryn A. Shelton, Michael A. Barry, K. Jagannadha Sastry

The sixth author's name is incorrect. The correct name is: Kathryn A. Shelton. The correct citation is: Dorta-Estremera S, Nehete PN, Yang G, He H, Nehete BP, Shelton KA, et al. (2017) Minimally invasive monitoring of CD4 T cells at multiple mucosal tissues after intranasal vaccination in rhesus macaques. PLoS ONE 12(12): e0188807. https://doi.org/10.1371/journal.pone.0188807
